# *Scleromitrion diffusum *(Willd.) R. J. Wang Inhibits Gastric Cancer via ERBB2/ERBB3/PI3K/AKT Pathway

**DOI:** 10.5152/tjg.2024.24152

**Published:** 2024-11-01

**Authors:** Wei Ye, Qiu Zhao, Peng Li, Tong Zhou

**Affiliations:** Department of Oncology, Changzhou Cancer Hospital, Changzhou, China

**Keywords:** *Scleromitrion diffusum* (Willd.) R. J. Wang, gastric cancer, network pharmacology, antitumor, PI3K/AKT pathway

## Abstract

**Background/Aims:**

This study aimed to evaluate the anticarcinogenic potential of *Scleromitrion diffusum* (Willd.) R. J. Wang (SD) extracts in vitro, along with exploring the underlying compatibility mechanisms.

**Materials and Methods:**

*Scleromitrion diffusum (Willd.) R. J. Wang* extract was prepared and gastric cancer (GC) cells were treated to detect the half maximal inhibitory concentration (IC50)/proliferation and migration/invasion by MTS method and transwell assay. The compatibility mechanisms of SD were analyzed by systems pharmacology strategy, combined with cellular experimental validation.

**Results:**

*Scleromitrion diffusum *(Willd.) R. J. Wang extract showed inhibitory ability on the proliferation of the GC cell lines dose- and time-dependently. A total of 3 active ingredients are involved in anti-gastric cancer effects of SD, based on the top 50 pathways. The “herb-composition-target-pathway” network showed the multi-target and multi-pathway characteristics of SD. There were 52 related targets shared by SD and GC. The cellular experiments supported that SD significantly reduced ERBB2 and ERBB3 expression levels in GC cells. The overexpression of ERBB2 or ERBB3 partially offset the anti-tumor effects of SD.

**Conclusion:**

*Scleromitrion diffusum* (Willd.) R. J. Wang inhibited gastric cancer growth and metastasis in vitro, which may be related to the inhibition of the ERBB2/ERBB3/PI3K/AKT pathway.

**Main Points**

- *Scleromitrion diffusum *(Willd.) R. J. Wang targets multiple genes and multi-pathway related to GC.
- *Scleromitrion diffusum *(Willd.) R. J. Wang inhibits GC growth and metastasis in vitro.
- *Scleromitrion diffusum *(Willd.) R. J. Wang inhibits the ERBB2/ERBB3/PI3K/AKT pathway.

## Introduction

Gastric cancer (GC) originates from epithelial tissue and is subordinate to one of the most common gastrointestinal tumors.^1^ There are nearly 2 million new cases of GC worldwide every year, with approximately two-thirds coming from developing countries and increasing year by year.^2,3^ Among the mortality rates of malignant tumors in China, GC ranks third in terms of mortality rate.^4^ The treatment of GC is mainly based on surgery, supplemented by radiotherapy, chemotherapy, or medication to form a comprehensive treatment.^5^ Specially, surgical treatment is generally suitable for the early stage.^6^ Chemotherapy is a common treatment for the advanced stage.^7^ However, most of the chemical drugs used for chemotherapy are artificially synthesized and have side effects.^8^ Even worse, some chemotherapy drugs have already developed resistance in GC, and the treatment effect is not ideal.^9^ Therefore, finding drugs with minimal toxic side effects and effective anti-tumor potential is the key to research.

Medicinal plants have gradually become the main research direction for screening anti-tumor drugs.^10^ *Scleromitrion diffusum* (Willd.) R. J. Wang (SD) (syn. *Hedyotis diffusa* (Willd.)) is a medicinal plant that has been used for a long time in China.^11^ It has the functions of protecting nerves and acting as an anti-inflammatory and antioxidant.^12,13^ The anti-inflammatory effect of SD on systemic lupus erythematosus targets STAT3, which involves the IL-6/STAT3 pathway.^14^ In China, SD is widely used for lung cancer treatment.^15^ SD is an important single herb for the therapy of advanced nasopharyngeal carcinoma.^11^ Compound Yangshe granule consists of SD and has anti-cervical cancer effects through the PI3K/AKT pathway.^16^ However, there are few reports on the therapeutic potential and the interaction of SD with the genome in human GC cells.

This study aimed to evaluate the anti-cancer capability of SD in vitro using GC cell lines, including anti-proliferation and anti-migration/invasion effects. Its mechanism of action was explored using network pharmacology and cell experiments.

## Materials and Methods

### Preparation of *Scleromitrion diffusum* (Willd.) R. J. Wang extract

*Scleromitrion diffusum* (Willd.) R. J. Wang herb was purchased from the North China Institute for Food and Drug Control (batch numbers 121183-201404 and 121183-201605, Beijing, China). The purchased *Scleromitrion diffusum* (Willd.) R. J. Wang was in dry whole grass powder form and had been tested by our pharmacy department to meet the quality standards specified in the Pharmacopoeia of the People’s Republic of China (2020). The 2 batches of *Scleromitrion diffusum* (Willd.) R. J. Wang powder were mixed and weighed for use. *Scleromitrion diffusum* (Willd.) R. J. Wang of the indicated weight was added to 10× volume ethanol (70%), heated, and refluxed at 100°C for 2 hours, for 3 repeats.^17^ Then, the extract solutions were filtered and concentrated, and the extracts were freeze-dried and stored at 4°C. The total content of flavonoids (1.86 g scutellarin equivalents/100 g SD) was determined using ultra-performance liquid chromatography/MS analysis to guarantee the quality of the extract.

### Cell Culture and Transfection

Human GC HGC-27 (HGC27, #TCHu 22) and AGS (#TCHu232) cell lines, from the Institute of Biochemistry and Cell Biology of CAS (Shanghai, China), were cultivated in RPMI 1640 (GIBCO, USA), fortified with 10% fetal bovine serum (FBS, Invitrogen, USA). HGC-27 and AGS cells were transfected with pcDNA3.1 plasmids carrying ERBB2/HER2 (ov-ERBB2) or ERBB3/HER3 (ov-ERBB3) sequence or negative control pcDNA3.1 vectors (ov-NC) using Lipofectamine 3000 reagent (Invitrogen, USA).

### Determination of Cell IC50

When human GC SGC-7901 cells reached the logarithmic growth phase, final concentrations of 0, 10, 30, 50, 70, and 90 μg/mL of SD were added for 24-hour, 48-hour, and 72-hour co-incubations. Then, the cells were collected for determination of cell viability using the MTS Assay Kit according to the Abcam (USA) instructions. The SD concentrations used for AGS cells were 0, 10, 20, 30, 40, and 60 μg/mL. The cell growth inhibition rates were calculated as follows: cell inhibitory rate = (1 − OD_490nm_ in the treated group/OD_490nm_ in the control group) × 100%.

### Determination of Cell Proliferation

The effects of SD on the proliferation of GC cell lines were detected by the MTS Assay Kit (Abcam, USA), according to the instructions.

### Determination of Cell Migration and Invasion

Cell migration was determined using Transwell chambers from Costar (Corning, Acton, MA, USA) with 8 µm pore size inserts, while cell invasion was detected by the Matrigel (Sigma Aldrich, St Louis, MO, USA)-coated chambers. The transwell system had 2 chambers separated with a membrane: the lower chamber contained 700 µL of medium containing the chemoattractant (15% FBS) and the upper chamber contained the cell suspension. The system was incubated for 24 hours. The cell number on the bottom (migrating cells) was detected under an inverted microscope.

### Network Pharmacology Analyses

Chemical ingredients of SD: the information on the active ingredients of SD was collected from TCMSP. Considering that SD is generally used orally in cancer treatment, we screened the potential active ingredients in SD using oral bioavailability (OB) ≥35% and drug-likeness (DL) ≥0.15.

Target prediction: the interactions between SD ingredients and the genome were inquired on TCMSP. The proteins targeted by SD were converted into gene IDs using the UniProt database conversion tool. The genes related to GC were collected from GeneCards, using the screening criteria of a Relevance score ≥30. The shared genes, a total of 52, between SD targets and GC-related genes were obtained using Venn diagrams.

Herb−ingredient−target−pathway network construction: The top 50 pathways enriched by the 52 shared genes were obtained using OmicShare tools. The genes and ingredients related to the top 50 pathways were traced. Network building of herb−ingredient−target−pathway interactions utilized Cytoscape 3.6.1 software.

### Analyses of mRNAs and Proteins

Quantitative real-time polymerase chain reaction: The total RNA used in this study was extracted with the help of the PureYield RNA Midiprep System (Promega, USA). The quality of the RNA was verified. cDNA was reverse transcribed from 3 μg of total RNA as described in the M-MLV Reverse Transcriptase kit (Invitrogen, USA). PCR reactions were run on a CFX96 Real-Time System (Bio-Rad, USA), under the use of Sso Advanced Universal SYBR Green Supermix (Bio-Rad, USA). The relative expression was calculated utilizing the 2^–ΔΔCt^ approach.

### Western Blot

Cell lysates were prepared using RIPA buffer (Millipore, USA) with the addition of a protease inhibitor cocktail (Roche, USA). Fifty micrograms of protein (pre-quantified with a Bradford assay) were heated in SDS sample buffer, centrifuged, and then loaded onto NuPage gels (Invitrogen, USA). After electrophoresis, the immunoblot PVDF membranes carrying proteins were blocked using PBST-5% BSA and incubated with antibodies directed against p-Akt, total-Akt, and beta-actin (Cell Signaling Technology, USA), each diluted 1:1000, overnight at 4°C, followed by incubation with a secondary horseradish peroxidase-conjugated antibody (Cell Signaling). Blots were visualized using the Thermo Scientific SuperSignal West Pico PLUS (Thermo Scientific, USA) and analyzed with NIH ImageJ densitometry software.

### Statistical Analysis

The data analysis results were expressed as mean ± SD. The statistical significance was determined when the *P*-value was less than .05. We used the Shapiro–Wilk normality test to evaluate the normality of data distribution. For normally distributed data, *t*-tests or 1-way analysis of variance (ANOVA) were used to analyze the differences between experimental groups, followed by post-hoc Duncan tests. When the data did not follow a normal distribution, the Kruskal–Wallis test was applied, followed by post-hoc Dunn’s tests.

## Results

### IC50s of *Scleromitrion diffusum* (Willd.) R. J. Wang for Gastric Cancer Cells

**Figure 1.**
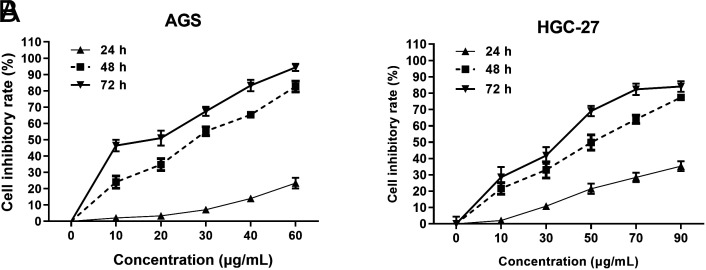
*Scleromitrion diffusum* (Willd.) R. J. Wang had an inhibitory effect on the growth of gastric cancer cells. (A) The growth inhibition curve of *Scleromitrion diffusum* (Willd.) R. J. Wang on AGS cells. (B) The growth inhibition curve of *Scleromitrion diffusum* (Willd.) R. J. Wang on HGC-27 cells.

The cell growth test results, as expected, showed that SD was able to inhibit the proliferation of GC AGS cells, with IC50s of 113.2 (95% confidence interval [CI]: 100.4-132.1), 25.97 (95% CI: 24.28-27.69), and 14.05 (95% CI: 11.53-16.41) μg/mL at 24, 48, and 72 hours, respectively (Figure 1A). Based on the proliferation inhibition rate of GC HGC-27 cells, the IC50 values of SD were calculated to be 139.5 μg/mL (95% CI: 126.7-156.9) for 24-hour treatment, 43.94 μg/mL (95% CI: 39.18-48.85) for 48-hour treatment, and 27.55 μg/mL (95% CI: 23.42-31.84) (Figure 1B). Therefore, the inhibition of SD on the growth of GC cells was concentration dependent.

### *Scleromitrion diffusum* (Willd.) R. J. Wang Inhibited the Function of Human Gastric Cancer Cells

**Figure 2.**
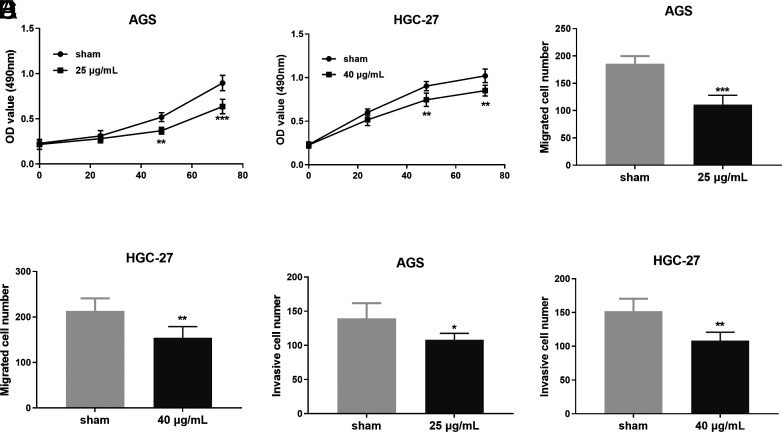
*Scleromitrion diffusum* (Willd.) R. J. Wang had inhibitory effects on the proliferation, migration, and invasion of gastric cancer cells. AGS (A) and HGC-27 (B) cells were treated with 25 μg/mL of *Scleromitrion diffusum* (Willd.) R. J. Wang extracts for 48 hours, and the cell proliferation was determined by MTS method. Transwell migration analysis of AGS (C) and HGC-27 (D) cells. Modified transwell invasion analysis of AGS (E) and HGC-27 (F) cells. ^*^ *P-*value <.05, ^**^ *P-*value <.01, ^***^ *P-*value <.001.

Then, we used 25 μg/mL SD to treat the AGS cell line and 40 μg/mL SD to treat the HGC-27 cell line to evaluate the action of SD on the proliferation, migration, and invasion ability of gastric cancer cells. As shown in Figure 2A and 2B, a time-dependent inhibition of cell viability after SD treatment was observed in AGS and HGC-27 human gastric cancer cell lines (*P-*value < .01). Therefore, we evaluated the abilities of these cells to invade and migrate in vitro. Following SD treatment at the dose of 25 μg/mL, AGS cells were weakened in metastatic abilities (*P*-value < .001, Figure 2C). For the HGC-27 human GC cell line, SD treatment reduced the migrated cell number (*P-*value < .01, Figure 2D). The ability of AGS aggressiveness in our results displayed a strong reduction in the presence of SD (*P-*value < .05, Figure 2E) compared to the control cells without SD treatment. Similar data were obtained for HGC-27 cell invasion ability (*P-*value < .01, Figure 2F).

### Network Pharmacology Analysis for *Scleromitrion diffusum* Against Gastric Cancer

**Figure 3.**
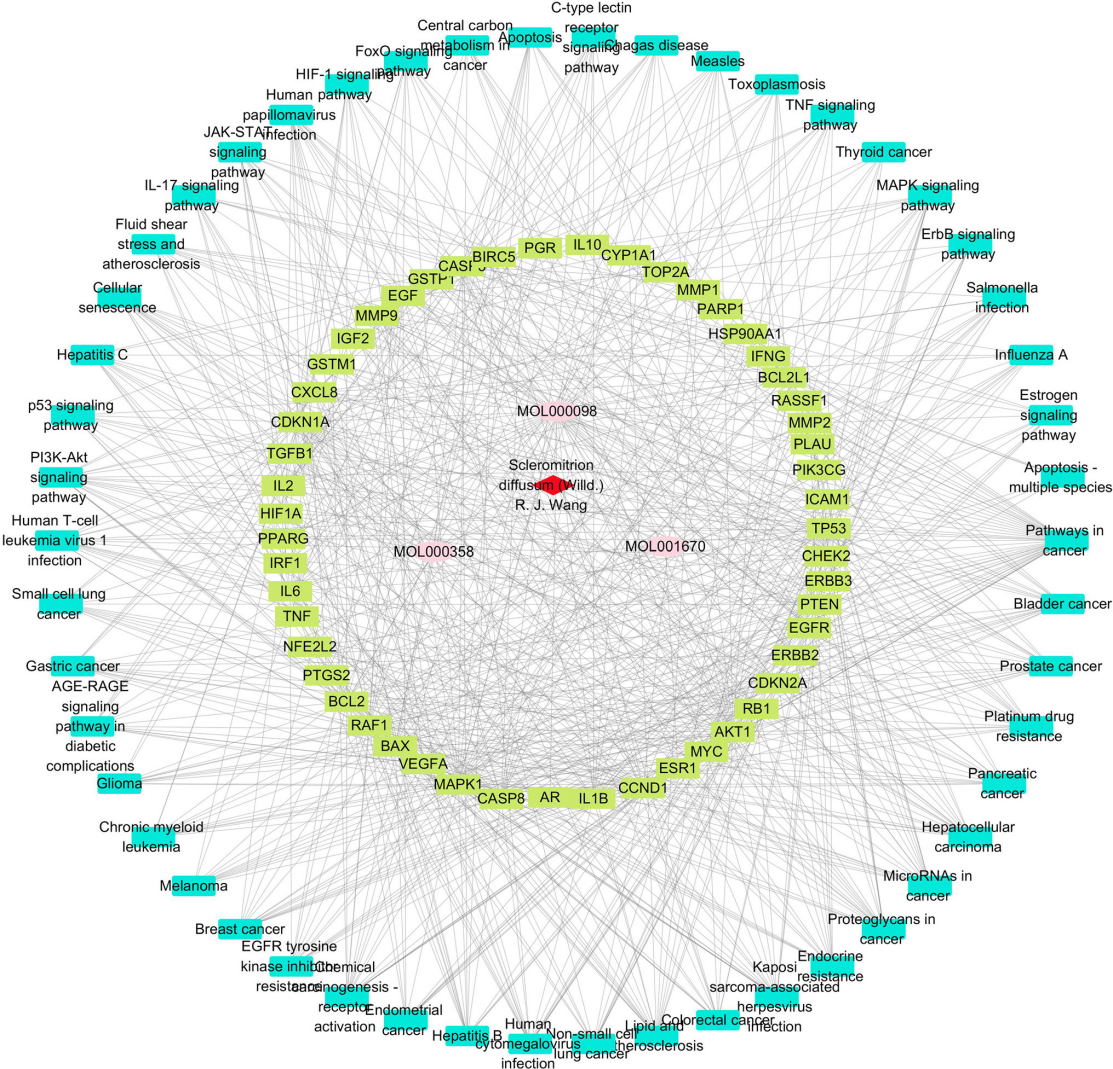
Construction of the “herb–composition–target–pathway” network of *Scleromitrion diffusum* (Willd.) R. J. Wang.

Based on the inquiries from TCMSP, a total of 37 pharmacologically active substances were collected from SD, among which 6 were pharmacologically active from a parameter perspective of OB (≥35%) and DL (≥0.15). According to the target-disease prediction system in TCMSP and GC-related genes in GeneCards, the top 50 pathways were predicted, and 52 genes were involved. Three active ingredients in SD were found to have potential effects against GC. The information on the 3 active components and related genes/pathways is shown in Figure 3. Then we chose ERBB2/ERBB3 and the PI3K/AKT pathway to validate.

### Effect of *Scleromitrion diffusum* on the Expression of ERBB2 and ERBB3 in Gastric Cancer Cells

**Figure 4.**
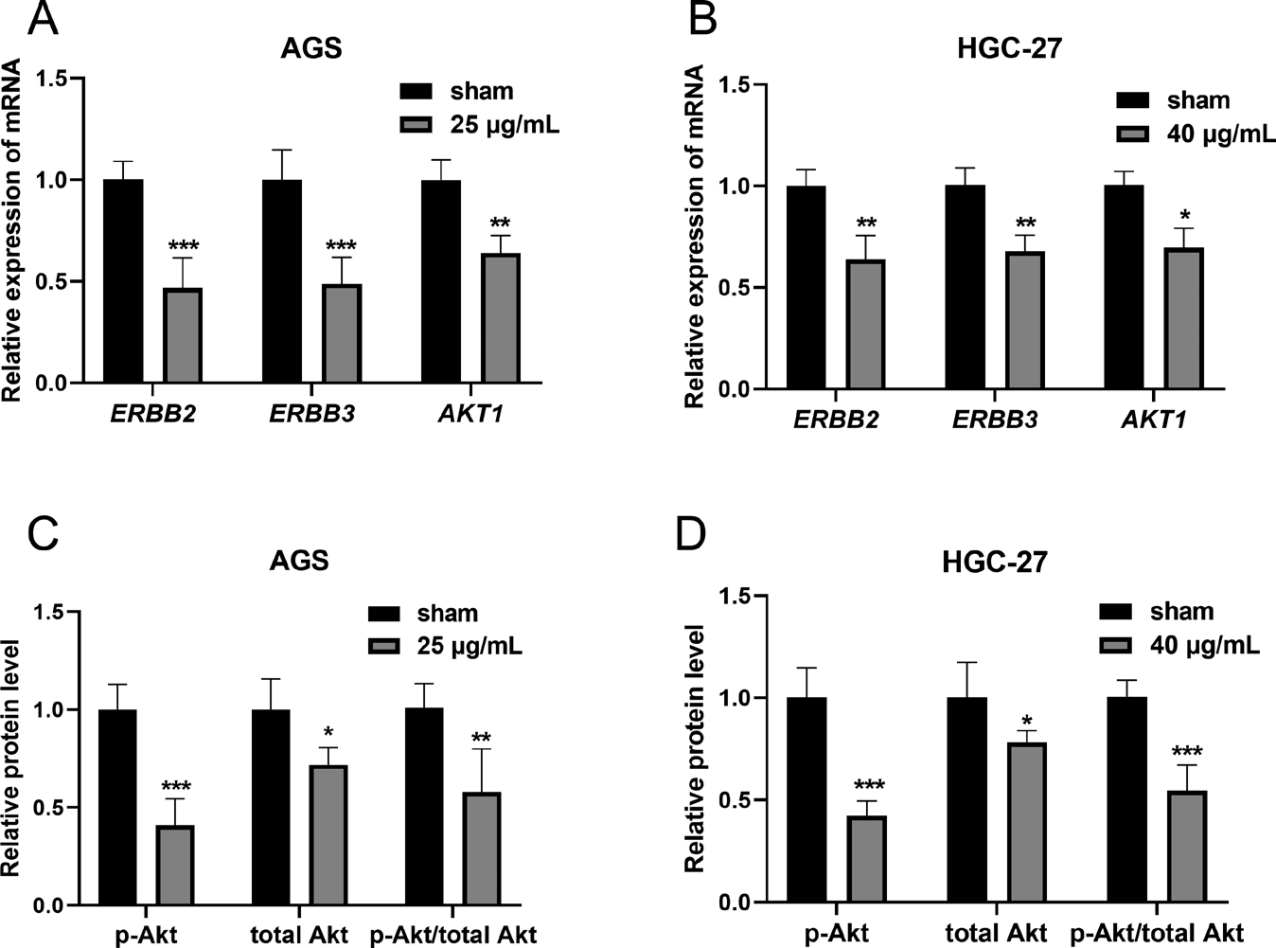
Changes in mRNA and protein expression in gastric cells treated with *Scleromitrion diffusum* (Willd.) R. J. Wang. (A) ERBB2, ERBB3, and AKT1 mRNA expression levels in AGS cells. (B) ERBB2, ERBB3, and AKT1 mRNA expression levels in HGC-27 cells. (C) p-Akt and total Akt protein expression levels in AGS cells. (D) p-Akt and total Akt protein expression levels in HGC-27 cells. ^*^ *P*-value <.05,^ **^ *P*-value <.01, ^***^ *P*-value <.001.

After treatment with 25 μg/mL SD for 24 hours, the expression levels of ERBB2 and ERBB3 mRNA were detected. Decreases in ERBB2 and ERBB3 mRNA levels were observed upon SD treatment, compared with those without SD treatment (*P*-value <.01, Figure 4A). Similar changes were also found in HGC-27 cells (*P*-value <.05, Figure 4B). Western blotting results showed a significant decrease in p-Akt and total Akt protein expression in AGS and HGC-27 cells treated with SD (*P*-value <.05, Figure 4C and 4D).

### Effect of *Scleromitrion diffusum* (Willd.) R. J. Wang in Gastric Cancer Cells under Overexpression of ERBB2 and ERBB3

**Figure 5.**
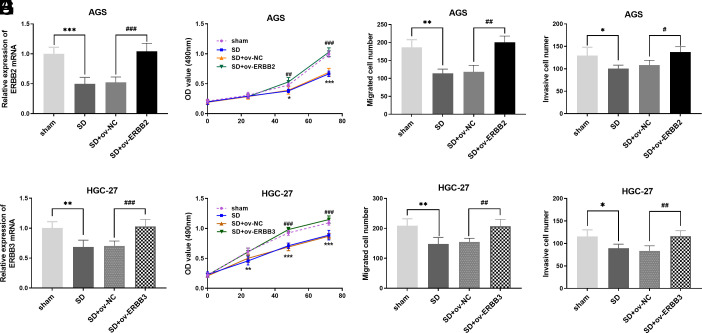
The influence of ERBB2/ERBB3 overexpression on the anticancer effect of SD (A) The ERBB2 expression in AGS cells after transfection. (B) AGS cell proliferation after ERBB2 overexpression. (C) AGS cell migration after ERBB2 overexpression. (D) AGS cell invasion after ERBB2 overexpression. (E) The ERBB3 expression in HGC-27 cells after transfection. (F) HGC-27 cell proliferation after ERBB3 overexpression. (G) HGC-27 cell migration after ERBB3 overexpression. (H) HGC-27 cell invasion after ERBB3 overexpression. ^*^ *P*-value <.05, ^**^ *P*-value <.01, ^***^ *P*-value <.001, vs. sham. ^#^ *P-*value <0.05, ^##^ *P*-value <.01, ^###^ *P-*value <.001, vs. SD+ov-NC. SD, *Scleromitrion diffusum* (Willd.) R. J. Wang.

The ERBB2−ERBB3 heterocomplex is emerging as important for the progression of GC.^18^ We then tested if overexpression of ERBB2 or ERBB3 weakens the anti-tumor effects of SD. Compared with the negative control, the expression of ERBB2 mRNA increased when cells were transfected with ERBB2 pcDNA (*P*-value <.001, Figure 5A). A cell proliferation assay showed that, compared with the negative control, cell proliferation increased when ERBB2 was overexpressed, even if under SD treatment (*P*-value <.001, Figure 5B). Also, overexpression of ERBB2 substantially abrogated the inhibition of migration (*P-*value <.01, Figure 5C) and invasion (*P-*value < .05, Figure 5D) by SD treatment. The ERBB3 plasmid successfully increased the expression of ERBB3 mRNA in HGC-27 (*P-* value <.01, Figure 5E). The effects of the ERBB3 plasmid were verified. The results suggested that the inhibitory effects of SD on HGC-27 proliferation (*P-*value <.01, Figure 5F), migration (*P-*value <.01, Figure 5G), and invasion (*P-*value <.05, Figure 5H) were substantially abrogated by overexpression of ERBB3.

## Discussion

This study made use of in vitro and network pharmacology methods to reveal the anti-carcinogenic potential of SD on the growth of human GC cells and the mechanisms by which SD might influence cellular function. We found that SD can inhibit the proliferation of GC cells in both dose-dependent and time-dependent manners. It also has an inhibitory effect on the migration and invasion of GC cells. After network pharmacology analysis, we found that SD can resist GC through multiple targets and pathways. After verification, we found that ERBB2 and ERBB3 are important targets for SD in GC, and of course, are involved in the downstream PI3K/AKT pathway.

Traditional Chinese medicine regards individuals as a system, accumulating a large amount of plant medicines and formulas. Cancer is a phenotypically and genetically heterogeneous disease that involves multiple genetic and epigenetic changes. The molecular characteristics of tumor tissue are crucial for effectively targeting cancer cells. The exploration of traditional Chinese medicine ingredients and targets will inject new vitality into the development of new drugs. Here, we identified quercetin, beta-sitosterol, and 2-methoxy-3-methyl-9,10-anthraquinone in SD as the active ingredients against GC. Quercetin is a well-established anti-GC chemical due to its pro-apoptotic, anti-proliferative, and anti-helicobacter activities.^19^ β-sitosterol has exhibited its potential to interfere with multiple cancer-related signaling pathways, including cell proliferation, survival, and invasion, without significant toxicity.^20^ 2-methoxy-3-methyl-9,10-anthraquinone has been previously identified as a key compound in the treatment of lung adenocarcinoma.^21^ All these previous findings are consistent with the findings in this study.

Previous cellular experiments showed that SD inhibited tumor growth when combined with *Scutellaria barbata* D. Don (SB), which might benefit from apoptosis induction via the EGFR/PPARγ/PI3K/AKT pathway.^17^ Here, we verified that SD can target ERBB2 and ERBB3 in GC cells. Gastric cancer harbors aberrations in the FGFR2/ErbB3/PI3K pathway or the activated ErbB2/PI3K pathway.^18^ Moreover, quercetin, targeting ERBB2/ERBB3, can inhibit colon cancer cell growth and induce apoptosis via the Akt pathway.^22^ This study used network pharmacology analysis and cellular experiments to discover that SD may inhibit the activation of the PI3K/AKT pathway and treat GC by acting on ERBB2 and ERBB3.

In summary, this study explored the inhibitory effect of SD on the malignant phenotype of GC cells, which may be due to SD targeting the ERBB2/ERBB3/PI3K/AKT pathway, using network pharmacology and cell experiments. This study provides new ideas for the treatment of GC.

## Data Availability

The data that support the findings of this study are available on request from the corresponding author.
